# Correlations of charge neutrality level with electronic structure and *p-d* hybridization

**DOI:** 10.1038/srep40843

**Published:** 2017-01-19

**Authors:** Arkaprava Das, Subodh K. Gautam, D. K. Shukla, Fouran Singh

**Affiliations:** 1 Inter University Accelerator Centre, Aruna Asaf Ali Marg, New Delhi-110067, India; 2 UGC-DAE Consortium for Scientific Research, University Campus, Khandwa Road, Indore 452017, India

## Abstract

The formation of charge neutrality level (CNL) in highly conducting Cadmium oxide (CdO) thin films is demonstarted by the observed variation in the band gap upon annealing and doping. It may be explained by the observation that Tin (Sn) doping breaks the perfect periodicity of CdO cubic crystal structure and creates virtual gap states (ViGS). The level of local CNL resides at the branch point of ViGS, making the energy at which native defect’s character changes from predominantly donor-like below CNL to predominantly acceptor-like above the CNL and a schematic band diagram is developed to substantiate the same. Further investigations using soft x-ray absorption spectroscopy (SXAS) at Oxygen and Cadmium edges show the reduction of Sn^4+^ to Sn^2+^. The analysis of the spectral features has revealed an evidence of *p*-*d* interaction between O 2*p* and Cd 4*d* orbitals that pushes the valence band minima at higher energies which is symmetry forbidden at г point and causing a positive valance band dispersion away from the zone centre in the г ~ L, K direction. Thus, origin of the CNL is attributed to the high density of the Oxygen vacancies as confirmed by the change in the local electronic structure and *p-d* hybridization of orbitals.

Cadmium oxide (CdO) is an n type degenerate semiconductor with direct band gap of 2.2 eV^1^ and non-stoichiometric composition due to the presence of Cd interstitials^2^ or oxygen vacancies^3^ which act as doubly charge donors^4^. It crystallizes in rocksalt phase and shows transparency almost in the whole visible region of the electromagnetic spectrum^1^. It has an intrinsic high electrical conductivity with high optical transmittance. CdO is a well known wide band gap semiconductor for exhibiting very high intrinsic mobility without additional doping to the lattice. Due to its distinguishable features mentioned above in recent years CdO has attracted the attention of research community. CdO is potentially an ideal transparent conducting oxide (TCO) for optoelectronic devices operating at lower wavelengths^5^ region, flat panel display^6^ and thin film photovoltaics. It can be used for full range photovoltaic applications with a pseudo alloy developed with ZnO. CdO is also useful for various versatile applications like transparent electrodes, gas sensors, liquid crystal display, photodiodes, and phototransistors. In order to enhance the electrical conductivity and band gap, Sn doping has been used as its ionic radii of Sn^4+^ is less than Cd^2+^ and electronegativity is little bit higher than Cd. So replacement of Cd ions by Sn ions is expected after doping.

Raman spectroscopy is one of the most prominent tools for the investigation and characterization of semiconductor material. First order Raman modes are forbidden for rocksalt structures like CdO. In the 1960s, zone centre transverse-optical (TO) and longitudinal-optical (LO) phonon modes of CdO were suggested to be located at 262 cm^−1^ and 523 cm^−1^ respectively by infrared measurements^7^. We performed Raman scattering on annealed thin film prepared by solgel method and find 3 main peaks i.e. one TO mode centred at 290 cm^−1^ and two LO modes centred at 472 cm^−1^ and at 952 cm^−1^. The dependence of Raman spectra on annealing temperature and doping have been discussed in detail. It is also known that the study of the hybridization of orbitals in CdO exhibits interesting phenomenon for the understanding the fundamental investigations of Physics. It may also be noted that the information about Bohr exciton radius of CdO is not well reported in the literature due to the peculiar behaviour of the band structure, which has been ascribed to the electronic structure. Therefore, the present manuscript is focused on the effect of crystallite size and doping on the electronic structure and is investigated within the frame work of charge neutrality level (CNL).

## Methods

Undoped and doped CdO films were deposited on the corning glass, silica and silicon substrates using sol-gel spin coating technique. Cadmium acetate dihydrate [Cd(CH_3_COO)_2_.2H_2_O] and Tin chloride pentahydrate were taken as a source of Cd and Sn in the solution. 2- Methoxy ethanol and Di-ethanolamine (DEA) were used as a solvent and sol stabilizer. In case of undoped solution the precursor was prepared with desired molarity by mixing Cadmium acetate dehydrate with 2- methoxy ethanol and stirred for 10 hours with the help of a magnetic stirrer. In case of 1% Sn doped CdO the precursor solution with intended molarity was prepared by mixing proper amount of Tin chloride pentahydrate with 2- methoxy ethanol. This precursor solution was stirred for 20 minutes. After that proper amount of Cadmium acetate dihydrate was added to the stirred solution which was stirred for 10 hours. Both the solutions were prepared at room temperature. For proper gelation, prepared solutions were aged for 2 days. As a precautionary measure the corning glass and silica substrate were rinsed in acetone and deionised water solution in an ultrasonic bath for 10 minutes. The silicon substrates were rinsed in 5% hydrofluoric acid solution (acid + deionised water) in an ultrasonic bath for 10 minutes and subsequently washed by deionised water to remove the oxidised layer. The films were deposited with the help of a spin coater with a speed of 2800 revolution per minute (rpm) for 30 seconds. The films were dried after each coating using a hot plate at 200 °C for evaporating organic residuals. This process was repeated for 12 times to get a homogeneous distributed film with desired thickness. The prepared undoped films were annealed at 400 and 500 °C in the oxygen environment respectively. The Sn doped films were annealed at 400 °C at oxygen environment. Annealing was done using a tubular furnace.

The structural characterization of Sn_x_Cd_1-x_O thin films was done by using Bruker High resolution X-Ray diffractometer system in the 2θ range 30°–70° with Cu K_α_ radiation with step size of 0.02°. The micro-Raman measurements were carried out using Renishaw InVia Raman microscope under the excitation by 514.5 nm argon ion laser operating at 50 mW of power. The thickness of films were determined using Rutherford Backscattering Spectrometry (RBS) using 2 MeV He^+^ ions as incident beam at back scattering detection angle of 165°. Surface morphology and compositional analysis of the films were investigated by Scanning Electron Microscopy (SEM) with MIRA\\TESCAN instrument using energy of 25 KeV. For recording transmittance and absorption spectra of the films, UV-Visible absorption measurements were carried out using Hitachi 3300 double beam spectrophotometer at room temperature. Surface topography measurements were carried out by atomic force microscopy (AFM) using NANAOSCOPE SPM II in tapping mode. The SXAS measurements of thin films at the O K and Cd M_4,5_ edges were carried out in total electron yield (TEY) mode at SXAS beam line (BL-01)of the Indus-2 synchrotron radiation source at Raja Ramanna Centre for Advanced Technology (RRCAT), India. For SXAS data processing Athena software package was used. These measurements were carried out in an ultra high vacuum ~ 10^−10^ Torr. The carrier concentration and Hall mobility were measured by Van der Pauw Ecopia HMS-3000 Hall measurement system.

## Results and Discussions

### Structural Properties

**Figure 1:**
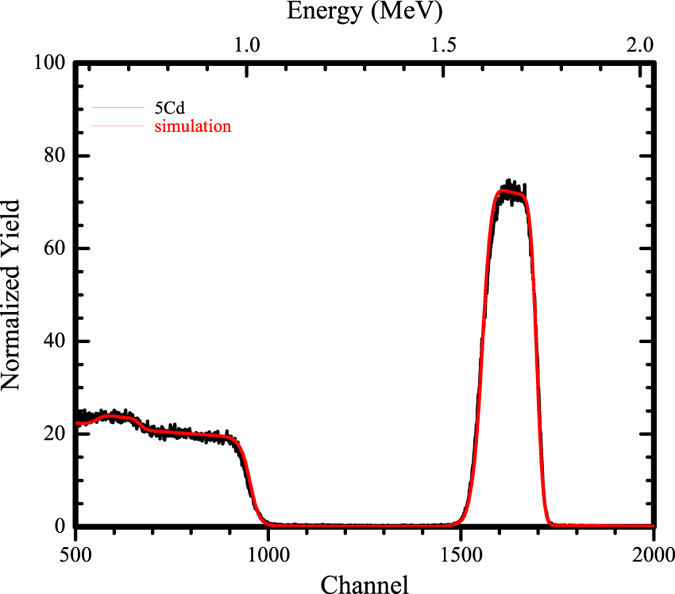
Rutherford backscattering spectrum for 5Cd thin film.

Pure CdO 500 °C annealed (5Cd) thin films were studied for their compositional analysis by RBS technique. The simulation was done by rump software. The thickness of the thin film was found to be 210 nm after simulation. The simulated spectrum of undoped CdO thin film had shown the proper stoichiometric ratio for the presence of Cd and O as their elemental atomic fraction was Cd-0.5 and O-0.5. However the interface between Si peak edge and lower energy edge of Cd is not very sharp. Fitting reveals a linear diffusion of Cd within the Si layer which is shown in Fig. 1. There is no significance of this simulation for Sn doped thin films as the kinematic factor of Sn and Cd is very nearby to each other^8^. So RBS technique can’t resolve those two elements.

**Figure 2:**
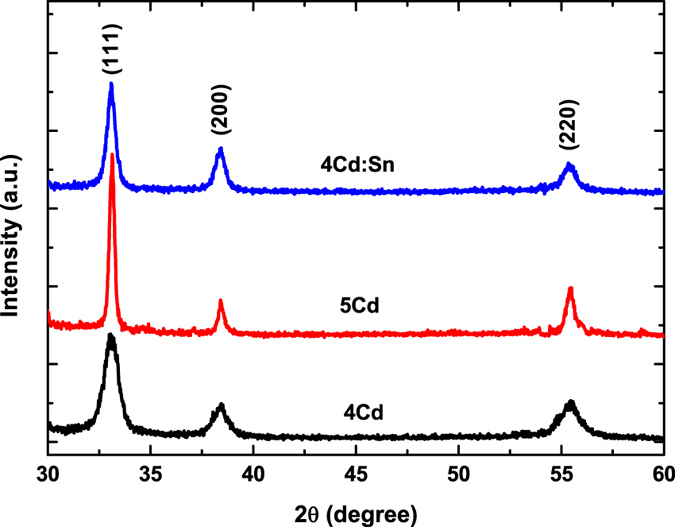
Grazing angle x-ray diffraction patterns for 4Cd, 5Cd, 4Cd:Sn thin films.

Figure 2 shows the glancing angle X-ray diffraction (GAXRD) pattern for undoped 400 °C (4Cd), 5Cd and 1% Tin doped 400 °C (4Cd:Sn) annealed CdO thin films. The GAXRD pattern reveals that CdO thin films are polycrystalline in nature and shows a cubic structure (JCPDS: 78–0653) at room temperature. The pattern shows that diffraction peak intensity corresponding to (111) plane has increased for undoped 5Cd and 4Cd:Sn film compared to 4Cd thin film which is a signature of particle diameter increment. Applying Scherrer equations to the full width at half maxima (FWHM) of the (111) peaks^9^,


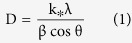


Where **λ** is the X-ray wavelength, θ is the Bragg’s diffraction angle (half of the peak position angle), β is the full width half maximum of the main peak in XRD pattern in radian. k is the shape factor generally its value is taken 0.9. Using this equation average particle diameter is determined for different thin films. The values of 2θ position and average particle diameter are cited in Table 1. There is almost no change in the 2θ value for the (111) peak for these thin films. The observed decrement in FWHM of (111) peak for 5Cd and 4Cd:Sn films compared to 4Cd film indicates the enhancement of average particle diameter. It is reported by B.J. Zheng *et al*.^10^ that with Tin doping particle diameters decrease. But in our case, Tin doping exhibits particle diameter enhancement. It is well known that Cd^2+^, Sn^4+^, Sn^2+^ ions have standard ionic radii of 0.97 Å, 0.71 Å, and 1.12 Å^11^, respectively. An increase in crystallite size is a signature of decrease of CdO stoichiometry by substitutional replacement of Cd with Sn. Each Cd^2+^ ions are substituted by Sn^2+^ ions with reduction of Sn^4+^ via creating oxygen vacancies in the lattice. This replacement enhances the electron concentration which is further verified by Hall measurement and optical studies. Ionic radius of the Sn^2+^ is greater than Cd^2+^. So the replacement results a lattice expansion in CdO crystals and as a consequence crystallite growth has been observed. It is also noteworthy that the substitution of Cd^2+^ by Sn^2+^ not only changes the bond length but also changes the short range order parameter which might be another possible reason to produce a change in micro-structural strain. This change in micro-structural strain can be corroborated to the crystallization of polycrystalline thin films^12^. However, the 2θ position of (111) plane for 1Sn thin film in the XRD pattern remains unchanged. It is clear from the GAXRD pattern of Tin doped film that no additional diffraction peak for SnO_2_ has been formed. This proves that doping amount (1%) in CdO lattice was within solubility limit. Moreover, SnO_2_ phase is reported to appear when doping limit is 7.6% or greater^10^.

### Growth mechanism of CdO nanocrystallites

Generally nanocrystalline formation in thin films is dominated by nuclei formation, growth, and coalescence processes. At lower annealing temperature small crystallites get sufficient kinetic energy for initiation of coalescence process. If the nanoparticles diameter is limited within 5–10 nm range, cluster migration process takes place at lower growth temperature^13^. Ostwald ripening and sintering mechanism are dominated within 500 °C–900 °C range^13^. For ZnO crystallites, the growth kinetics is well explained by Wagner theory of Ostwald ripening^14^. Here coalescence process is expected to be dominated by cluster migration as the annealing temperature had not gone beyond 500 °C. It is well known that various kinds of defects are produced during the preparation of the thin films which induce stress in the films. During higher calcination temperatures the atoms in the lattice gain sufficient energy and get enough time to adjust their positions. As a consequence of that adjustment stress within the lattice tends to get relaxed.

**Figure 3:**
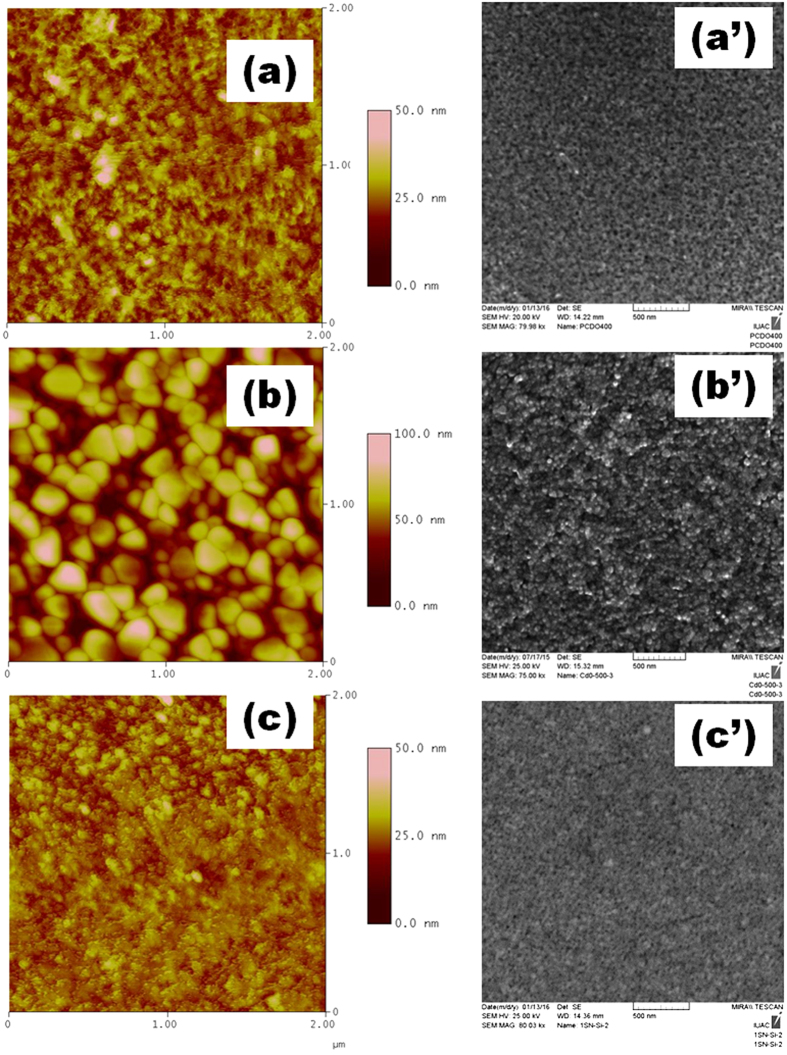
AFM and SEM images for 4Cd (a, a’), 5Cd (b, b’) and, 4Cd:Sn (c, c’) thin films.

From optoelectronic device application point of view, the surface properties of transparent conducting oxide influences the efficiency of the device and become a significant factor during device fabrication. In Fig. 3(a,b,c), (a’,b’,c’) the AFM and SEM images of 4Cd, 5Cd and 4Cd:Sn thin films are presented. All the images show uniformity in thin film without any huge crack or dust particle. 4Cd:Sn and 4Cd thin films are feebly crystallized and no voids are found. From the SEM images it is clear that with increasing annealing temperature, crystalline quality of the thin film enhances. This increment in average grain size with annealing temperature is further clarified by GAXRD pattern. This also results a decreases of the overall grain boundary area. The increment in root mean square (rms) roughness (calculated by nanoscope software) also verifies the decrease in grain boundary area which subsequently reduce the grain boundary scattering. The values of rms roughness and particle diameter are given in Table 1. The increment in *rms* roughness may lead to an enhancement of propagation loss for surface acoustic wave (SAW)^10^ and degrade the efficiency of photovoltaic solar cells. Therefore, the increment in grain size and surface roughness may have opposite influences on the electrical and optical properties. There is not much change in roughness between 4Cd:Sn and 4Cd thin films which means that there is not much change in the grain boundary area. Here, the roughness is in few nm, so a little bit of change in the roughness would have very limited impact on the propagation of SAW.

### Electrical properties

**Figure 4:**
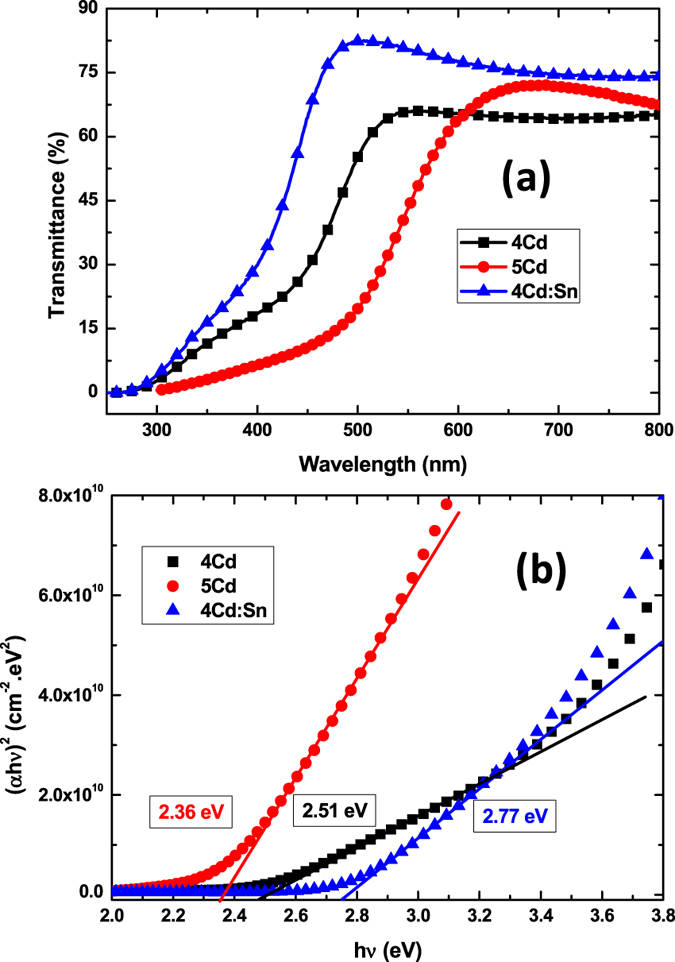
Transmittance spectra (a) and Tauc plot (b) for band gap calculation for 4Cd, 5Cd, and 4Cd:Sn thin films.

Hall measurement was carried out for the thin films in van der Pauw configuration at room temperature. The conductivity is reduced and carrier concentration increased drastically for Tin doped film. As mentioned earlier, each Cd^2+^ ion is substituted by a Sn^2+^ ion with reduction of Sn^4+^ via creating oxygen vacancies in the lattice which further enhances the carrier concentration and reduces the conductivity. It is quite clear that due to the substitution of Cd^2+^ by Sn^4+^, additional Oxygen vacancies are created at electrically active sites which liberate electrons and Sn^4+^ becomes Sn^2+^ in order to maintain the charge neutrality inside the lattice. The bulk carrier concentration of undoped 5Cd thin film is lower as-compared to the undoped 4Cd thin film. Due to higher annealing temperature in oxygen environment there might be a reduction in the concentration of Oxygen vacancies and Cd interstitials which is also observed from the sharper band edge in Tauc plot as shown in Fig. 4(b). This is a possible reason for reduction in carrier concentration in undoped 5Cd thin film. Enhancement in grain size improves the overall crystalline quality. An overall decrease in grain boundary fraction in the thin film reduces the grain boundary scattering which further reduces the resistivity. With Sn doping lattice distortion increases in CdO crystal subsequently an increase in grain boundary defects occurs. These defects produce scattering with the charge carriers which further reduces the mobility and conductivity of 1Sn thin film^10^. The values of resistivity and mobility are cited in Table 1.

From the above discussion it can be concluded that Tin would be an excellent choice as dopant in CdO for modifications of electrical properties as Sn can replace the Cd and enhances the carrier concentration which further reduces the conductivity but the doping concentration should be within the solubility limit which is about 4.4%. This excellent property with Sn doping can be exploited in the formation of oxide based optoelectronic device^15^.

### Optical properties

The transmittance spectra are shown in Fig. 4(a). The transmittances for all the thin films are above 60% for wavelength more than 600 nm to 800 nm. A prominent change in average transmittance has been observed in the 300 nm to 600 nm wavelength region. The transmittance in the absence of interference fringes is to be understood by Pankove analysis^16^. The transmittance in the visible region is found to vary from 55% to 75%. The 5Cd thin film shows a lower percentage of transmission and sharper band edge in Tauc plot in Fig. 4(b) caused by the decrease in Oxygen vacancies with increasing annealing temperature. The red shift and the blue shift of the cut off wavelength in the transmittance spectra denotes a band gap shortening and band gap widening compared with the 4Cd thin film. The undoped thin film became reddish from yellowish nature with increasing annealing temperature. The energy band gaps of the thin films are calculated from the absorption spectra using Tauc plot shown in Fig. 4(b). If we want to correlate the band gap variation with the particle diameter via quantum confinement effect, the results are not compatible. The dependence of absorption coefficient α on the photon energy (hν) is fitted using the relation^17^:


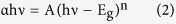


where A is a constant, hν is the photon energy, E_g_ is the optical band gap of the thin film to be calculated. Here the exponent n depends on the type of allowed transition in the material. It has value equal to ½, 2, 3/2 for allowed transitions, indirect transitions, and direct forbidden transitions respectively. CdO is direct energy band gap materials^18^. The extrapolation of the linear plot provides the value of band gap for direct allowed transition. The optical band gap values of thin films are found to be 2.51 eV, 2.36 eV, 2.77 eV for 4Cd, 5Cd, 4Cd:Sn thin films, respectively. The errors in the band gap calculations are at third place of the decimal point (+/−0.002 eV).

Carrier concentrations are different for these three samples and this can be attributed to the band gap variation through Burstein-Moss Shift (BMS)^19^,^20^. The observed higher band gap value for the Tin doped film comes from the filling of lower states of conduction band which is a common phenomenon for degenerate doped semiconductor. Due to BMS the enhanced value of the band gap is given by the following equation:


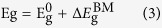


where E_g_° is the band gap of single crystal CdO and BMS is given by:


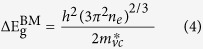


and


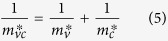


where E_g_°, 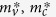
and 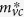 are the intrinsic band gap of an undoped semiconductor, the valence band effective mass, the conduction band effective mass and the reduced effective mass, respectively. The ratio of reduced effective mass to free electron mass 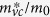 for pure CdO system is 0.274^21^ and the band gap of pure CdO system is 2.22 eV. It is noteworthy that the Bohr exciton radius for CdO is not reported in the literature so one can’t draw any quantitative conclusion regarding the correlation of effective mass for CdO and its crystallite size. Using the equation (4) BMS for each thin film is calculated. The calculated values of BMS are cited in Table 1 but those values are not quantitatively compatible for all three thin films with the actual value of variation of band gap which come from the Tauc plot. Band gap enhancement for 5Cd compared to pure CdO band gap value is almost equal with the BMS i.e. 0.11 eV. However, the band gap of 4Cd and 4Cd:Sn is enhanced by 0.29 eV and 0.55 eV, respectively as compared to the bulk CdO. Interestingly, for the 4Cd films a blue shift of 0.13 eV is taken care by BMS and 0.16 eV still remains. Similarly, for 4Cd:Sn the band gap has been enhanced by 0.55 eV, within that only 0.23 eV shift is taken care by BMS and 0.32 eV still remains, which may not be explained by particle size effect as it is increased considerably as compared to 4Cd thin film.

Further, the band gap widening can be explained in the framework of charge neutrality level (CNL). CNL can be identified as the demarcation between the surface states that are predominantly donor-like (below) and acceptor-like (above)^22^. Due to Sn doping perfect periodicity of CdO cubic crystal structure has been broken as Sn^4+^ ions would pretend to behave as native donor type defect which is attributed to the generation of virtual gap states (ViGS). The level of local CNL resides at the branch point of ViGS, making the energy at which native defect’s character changes from predominantly donor-like (valence band character) below CNL to predominantly acceptor-like (conduction band character) above the CNL^23^. So donor like ViGS are the surface states which reside below the local CNL. Due to prominent electronegativity and size mismatch between O and Cd atoms in CdO lattice the conduction band minima (CBM) at the г point is significantly lower compared to the rest of the Brillouin zone^24^. Further, *p*-*d* interaction between O 2*p* and Cd 4*d* orbitals pushes the valence band minima (VBM) at higher energies which is symmetry forbidden at г causing a positive valence band dispersion away from the zone centre in the г ~ L, K direction^25^. This explains well the reason for local CNL to reside intrinsically above CBM in CdO. So the donorlike ViGS below CNL from where each Sn^2+^ ion becomes Sn^4+^ by producing oxygen vacancies and the electrons from oxygen vacancies reach directly to the ViGS of conduction band as CNL lies above CBM leading to a large accumulation of electrons at the surface. Surface electron accumulation phenomenon in CdO has been well reported by King *et al*.^26^ Within the framework of amphoteric defect model the formation of donor type defects are more favourable when Fermi level is below compared to an energy level known as Fermi level stabilization energy (E_FS_)^27^. It is argued that there is an energy associated with each semiconductor which plays a role analogous to Fermi energy in metal^28^ and that energy is called CNL E_B_ for complex band structure. Intrinsic tendency of Fermi level for any semiconductor system is to get coincident with E_FS_ and E_FS_ tends to follow CNL as the system goes into the saturation. Correlation of E_FS_ and CNL is experimentally reported for GaAs by W. Walukiewicz^29^ and it correlates well with each other. It has been reported that CNL synonymously called midgap energy. It has been postulated as a reference point for pinning the Fermi energy at metal induced gap states which verifies our early discussion. From this postulate we can enunciate that in our case for 1Sn thin film virtual gap states have been generated due to incorporation of Sn and Fermi level pins taking CNL as reference energy level.

**Figure 5:**
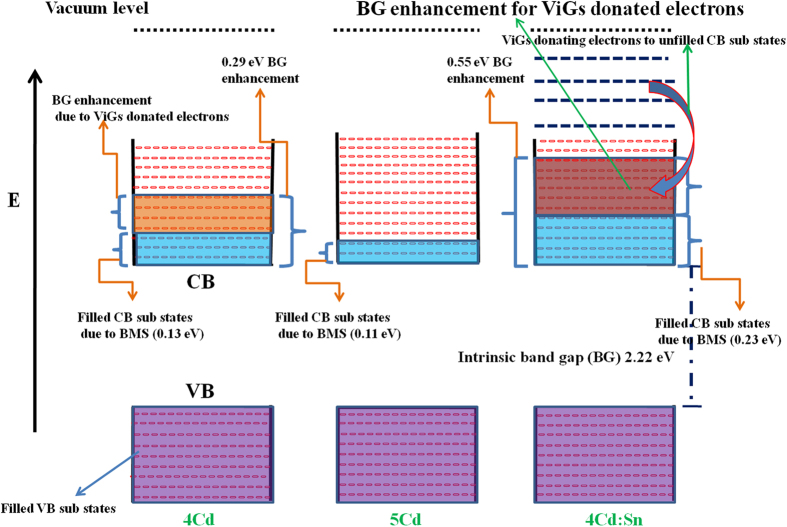
Developed schematic energy band diagram.

In covalent or semi-ionic semiconductor like CdO the coupling due to hybridization opens the optical band gap separating bonding from anti-bonding states. Incorporation of defects into the crystal drags bonding and anti-bonding states towards CNL which subsequently increase the density of states around CNL^30^. If E_FS_ coincides with CNL, the formation energy of donor type defects and acceptor type defects become equal and further change in the carrier density will not be possible by creating native defects. This means that the system goes to the saturation condition as further creation of donor or acceptor type defects is not possible by creating additional defects or doping into the lattice. Thus, E_FS_ favours the pinning of surface Fermi level above the CNL and resulting negative surface charge from occupied acceptor surface states which were unoccupied earlier when surface Fermi level was below than CNL. Due to overall alignment between surface and bulk region the Fermi level shifts little above CNL. As a consequence, for maintaining local charge neutrality there is an upward bending of the bands relative to the Fermi level and electron starts to deplete at the surface subsequently these surface depleted electrons fills the ViGS above CBM which explains the effective further band gap enhancement. This behaviour is well reported for Sn doped In_2_O_3_ by King *et al*.^31^. Band gap enhancement is shown schematically in the energy band diagram in Fig. 5. It is very difficult to depict the actual position of surface Fermi level, so in the energy band diagram the position of surface Fermi level has not been shown.

### Raman spectroscopy

**Figure 6:**
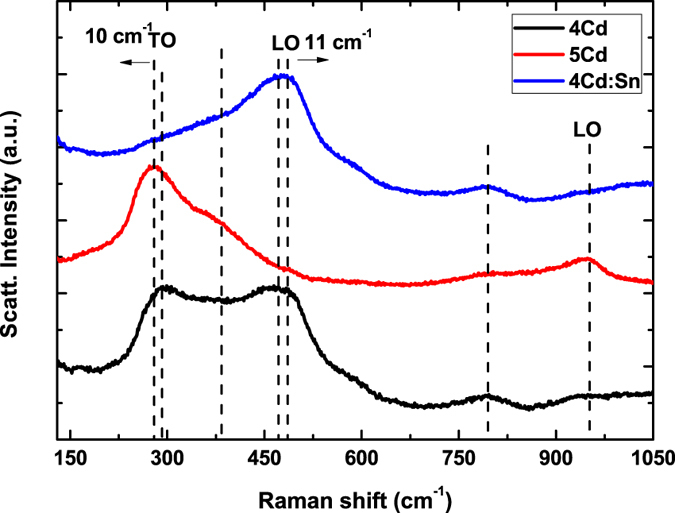
Raman spectra for 4Cd, 5Cd, and 4Cd:Sn thin films.

The space group symmetry of CdO rocksalt structure is Fm
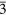
m. It is well established that A1, E1 both are Raman and IR active branches, there symmetries are polar, doubly degenerated and split into TO and LO components with different frequencies. E_2_(H) and E_2_(L) branches are non polar so they are Raman as well as IR inactive. Raman experiment was done in backscattering geometry, without any polarization analysis. The main features in Raman spectra have been shown Fig. 6. For 4Cd thin film a sharp peak is appearing centred at 290 cm^−1^, and two weaker bands are located at 793 cm^−1^, 952 cm^−1^ respectively. A broad spanning structure from 300 cm^−1^ to 500 cm^−1^ is observed. Within that range a sharper intense peak centred at 472 cm^−1^ is observed. Theoretically it has been reported that CdO molecules have LO and TO phonon modes at 478(25) cm^−1^ and at 262(3) cm^−1^, respectively^32^,^33^. It is also well reported that peak centred at 952 cm^−1^ is LO phonon mode. Generally, in CdO rocksalt structure only second order Raman scattering is expected. The excitation wavelength for the Ar laser was 514.5 nm which is close to the band gap of CdO so the probability of first order Raman scattering can’t be ruled out completely. It is well reported that the resonance effects are not important in determining the Raman spectrum line shape. According to the Raman selection rules for CdO rocksalt structure both LO and TO modes are dipole forbidden. All features over here in the Raman spectra can be attributed to the second-order scattering process in CdO^34^. The polarization vectors of incident and scattered light are parallel here, henceforth only E_2_ and A_1_ (LO and TO) modes would be allowed according to the Raman selection rule. The vibration of Cd and O sub-lattices are related to the E_2_ (high) and E_2_ (low) modes which are nonpolar and sensitive to strain but not influenced by the carriers or electric field of the crystal^35^.

For 5Cd thin film, we observe that 290 cm^−1^ TO mode and 952 cm^−1^ LO mode is getting sharper, 290 cm^−1^ TO mode shows a red shift about 10 cm^−1^, a weak sub peak around 370 cm^−1^ has been evolved, the subpeak centred at 793 cm^−1^ becomes more intense without any shift in peak position and the LO Raman peak centred at 472 cm^−1^ smears out completely. For 4Cd:Sn thin film, LO phonon mode centred at 472 cm^−1^ becomes sharper and shows a blue shift of 11 cm^−1^ but the TO phonon mode centred at 290 cm^−1^ smears out completely. The weaker sub-peak centred at 793 cm^−1^ sustains well in this case. It is reported that 290 cm^−1^ TO phonon mode is a defect incorporated Raman peak for CdO rocksalt phase^36^. It is redundant to say that lot of impurities are present during thin film preparation which produces stress in the lattice and with annealing at higher temperature the atoms in the lattice get sufficient time and energy to readjust their positions. As a consequence stress tends to get relaxed. For 5Cd thin film impurities and defects decrease compared to 4Cd thin film. This is favourable for CdO rocksalt phase to get more stable and the 290 cm^−1^ TO phonon mode for getting more prominent and intense in 5Cd thin film. 10 cm^−1^ red shift is a consequence of the relaxed lattice strain which can be called as softening of Raman phonon modes. Reducing band gap with increasing annealing temperature giving rise to a decrement in absorption coefficients for both excited and scattered radiation. This might be another possible reason for the sharper Raman peak at centred at 280 cm^−1^ for 5 Cd thin film.

During Raman measurement the incident light was perpendicular to the surface of the thin films; hence the presence of LO phonon modes is quite obvious. For 4Cd:Sn thin film it is already enunciated that Sn^2+^ ions replaces the Cd^2+^ ions in the lattice which might act as the impurity centre and breaks the translational symmetry of the CdO lattice. As a consequence, q = 0 Raman selection rule is relaxed and phonons with *q* ≠ 0 (q is the wave vector) contribute to the Raman scattering away from the zone centre. In other words this relaxation in wave vector results in scattering by phonons in the host matrix which have wave vector far from the zone centre^37^. Subsequently this leads to an asymmetric broadening and frequency shifts in phonon modes^38^. LO phonon modes of CdO results an atomic displacement along c-axis. The frequency shifts are proportionally related to the short range order parameter along c-axis, which is the distance between Cd and O atoms for pure CdO lattice. After replacement of Cd^2+^ ion by Sn^2+^ ion change in bond length as well as the short range order parameter takes place which deform the lattice and modulate the wave functions through deformation–potential scattering process^36^. Therefore a complex interaction mechanism takes place here which may speculate the observed 11 cm^−1^ blue shift of LO phonon mode centred at 472 cm^−1^. This can be enunciated as the stiffening of that phonon mode.

### Soft x-ray absorption spectroscopy (SXAS) studies

**Figure 7:**
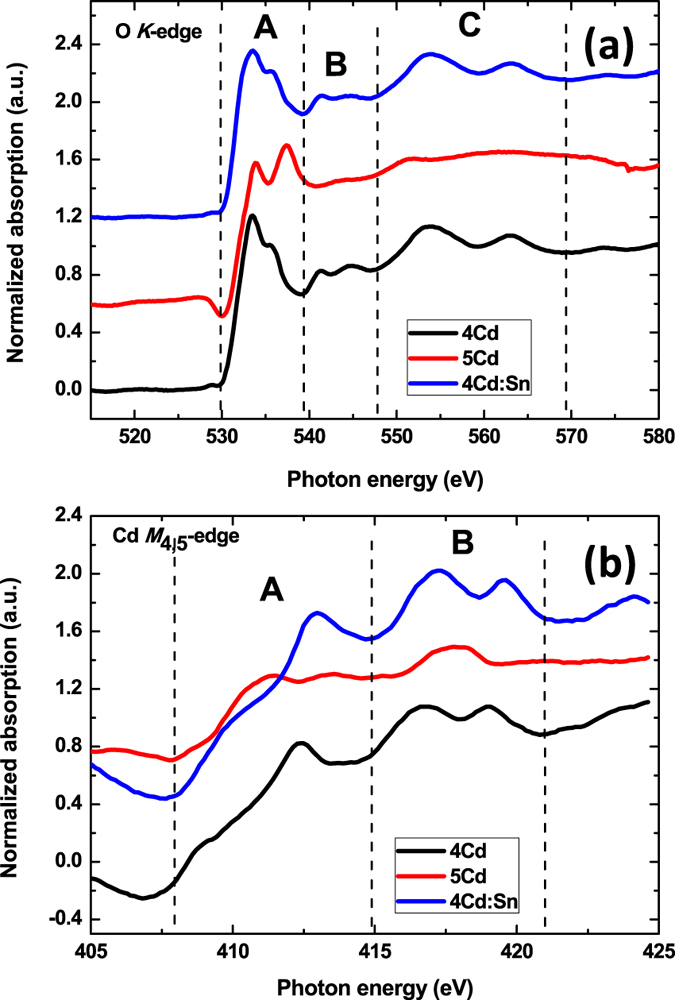
Soft X-ray absorption spectra for O k edge (a) and Cd M_4,5_ edge (b) for 4Cd, 5Cd, 4Cd:Sn thin films.

Soft X ray absorption spectra are recorded in TEY mode which has been measured from sample drain current. Figure 7 represents the experimental SXAS data for the Oxygen K edge and Cd M_4,5_ edge (normalized μ(E) versus photon energy (eV) of 4Cd, 5Cd, 4Cd:Sn thin films. Using ATHENA XAS data processing software (FEFF 6.0 code) the data were normalized and fitted to get the peak intensity ratio for Oxygen K edge which are cited in Table 2.

During Oxygen K edge peak fitting three different functions are taken i.e. arctangent, Gaussian, pseudo-voigt. From the fitting of 4Cd:Sn film it is observed that the centre of the arctangent function shifts by 840 meV below with respect to 4Cd thin film. This is direct indication that the CBM seems to be more towards Fermi level for 4Cd:Sn films and thus in agreement with our previous discussion that Fermi level shifting in case of 4Cd:Sn thin film above E_FS_. A higher electron concentration for 4Cd:Sn thin film also implies that conduction band is highly degenerate with a change in their effective mass. Henceforth the Fermi level is expected to be above conduction band and also to be pinned above CNL to maintain local charge neutrality. Moreover, the change in peak intensity ratio for 4Cd:Sn as compared to 4Cd thin film can be attributed to the pinning of Fermi level above CNL for surface electron accumulation phenomena. After replacement of Cd^2+^ ion by Sn^2+^ ion there is a change in the bond length due to different electronegativity and ionic radii as well as in the crystal field which might be also a possible reason for the change in peak intensity ratio. There is not much change in the overall spectral profile for 4Cd and 4Cd:Sn thin films. From this observation we can conclude that oxygen coordination in the neighbourhood of Cd or Sn atoms are more or less same which is also a signature of almost equal amount native defects namely Oxygen vacancies for both the cases.

For the case of 5Cd, a significant change in the peak intensity ratio as well as in the overall spectral profile for both Oxygen K and Cadmium M_4,5_ edges has been observed. It is well reported by *I.N. Demchenko et al*. that vacancies created into the Oxygen atom position has a deep impact on the overall spectral profile and edge shape^39^. Mainly spectral profiles are influenced by the native defects of first and second coordination shell. The change in spectral profile with increasing Oxygen vacancies has been theoretically well calculated by that group. Due to annealing at 500 °C in Oxygen environment CdO lattice gets sufficient time to readjust their atomic positions which releases stress and the number of Oxygen vacancies also reduces as Oxygen diffuses inside the system. Therefore the spectrum of 5Cd looks like the profile where Oxygen vacancies are very less and due to limited resolution, S and E characters cannot be observed^39^. Hence, the changes in the intensity ratio of two first resonance and overall spectral profile can be attributed to the reduced number of Oxygen vacancies for 5Cd thin film. The same signature is obtained also from Cadmium M_4,5_ spectral profile.

The dipole transitions from Oxygen 1 s core state to unoccupied state with p character above Fermi level are allowed. In case of a purely ionic Cd^2+^ O^2−^ bonding, the 2*p* of Oxygen state must be occupied and the transition from 1*s* to 2*p* should not be observed. However, those transitions are experimentally visible which is a clear signature that CdO thin film is not purely ionic but of a mixed character^24^,^40^. Cd_M4, 5_ edges also gives the same signature that CdO thin films are of mixed character. The d electrons of Cd have effect not only on the band gap but also on the ground state properties such as equilibrium lattice parameter and cohesive energies^41^,^42^,^43^. The interaction between Cd 4*d* and O 2*p* determines the type of band gap of CdO.

Particularly in CdO VBM is not at the centre of the Brillouin zone which leads to an indirect band gap contribution. This indirect band gap can be speculated to be the direct consequence of the hybridization of Cd 4*d* with O 2*p* states combined with octahedral point symmetry^25^. The hybridization of O 2*p* states with Cd 4*d* shallow electrons is well reported by Piper *et al*.^24^ by Xray emission spectroscopy (XES) which also supported by Xray photoelectron spectroscopy (XPS)^39^. The signature of O 2*p* hybridization with Cd 4*d* states is not as sharp as reported by the theoretical calculations elsewhere for the XAS spectra^39^. We can speculate that the practical reason behind this difference between theoretical and experimental spectra would be the polycrystalline nature of thin films. Same signature of mixing can also be obtained from Cd_M4, 5_ edges. For Oxygen k edges the peaks of the B and C region can be attributed to the interference effect of the multiple scattering signals. It is well reported from theoretical calculation by Demchenko *et al*.^39^ that with increasing cluster size there would be no generation of any new feature in the spectral profile apart from a little bit variations in the previously existing peaks for lower coordination.

## Conclusion

In summary, the formations the virtual gap states as charge neutrality level are reported and its correlations with electronic structure have been discussed. This level is the energy at which native defect’s character changes from donor-like to acceptor-like and it is well demonstrated by developing a schematic band diagram. Electronic structure using the soft x-rays absorption spectroscopy at the Oxygen and Cadmium edge has been investigated and it has revealed the reduction of Sn^4+^ to Sn^2+^ and its effect on the variation in the mobility of charge carriers. Interestingly, it is observed that the native defects like Oxygen vacancy has a prominent effect on the spectral features of Oxygen K edge and Cadmium M_4, 5_ edge gives an evidence of O 2*p*-Cd 4*d* hybridization. The growth of crystallites is understood by the expansion of lattice as results of replacement of Cd^2+^ ion by Sn^2+^ and liberation of electrons in the conduction band results an enhancement in band gap as well as in carrier concentration which also reduces the mobility. Thus, the present manuscript reports formation of charge neutrality level and its correlation with electronic structure and *p-d* hybridization is reported which has very interesting ramifications besides its crucial role for the development of optoelectronic devices.

## Additional Information

**How to cite this article:** Das, A. *et al*. Correlations of charge neutrality level with electronic structure and *p-d* hybridization. *Sci. Rep.* **7**, 40843; doi: 10.1038/srep40843 (2017).

**Publisher's note:** Springer Nature remains neutral with regard to jurisdictional claims in published maps and institutional affiliations.

## Figures and Tables

**Table 1:** Numerical values of different characterizations.

Features	400 CdO	500 CdO	1Sn
Particle diameter	8 nm	27 nm	17 nm
2θ position (degree)	33.14	33.13	33.22
mobility (cm^2^/V-s)	39.25	172.2	7.52
Band gap	2.51 eV	2.36 eV	2.77 eV
Conductivity (mho-cm^−1^)	1.41e02	9.101e02	8.80e01
*rms* roughness	4 nm	15 nm	3 nm
Bulk carrier concentration (cm^−3^)	2.845e19	2.25e19	7.30e19
Resistivity (ohm-cm)	5.78e-3	7.06e-03	1.13e-02
Calculated BMS	0.13 eV	0.11 eV	0.23 eV

**Table 2:** Peak intensity ratios from SXAS analysis.

Sample	First peak	Second peak	Intensity ratio (2^nd^/1^st^)
4Cd (O k edge)	1.197	0.747	0.62
4Cd:Sn(O k edge)	1.066	0.963	0.90
5Cd (O k edge)	0.555	1.086	1.96
